# Population Exposure Changes to Mean and Extreme Climate Events Over Pakistan and Associated Mechanisms

**DOI:** 10.1029/2023GH000887

**Published:** 2023-10-25

**Authors:** Farhan Saleem, Wenxia Zhang, Saadia Hina, Xiaodong Zeng, Irfan Ullah, Tehmina Bibi, Dike Victor Nnamdi

**Affiliations:** ^1^ International Center for Climate and Environment Sciences Institute of Atmospheric Physics Chinese Academy of Sciences Beijing PR China; ^2^ College of Earth and Planetary Sciences University of Chinese Academy of Sciences Beijing PR China; ^3^ State Key Laboratory of Numerical Modelling for Atmospheric Sciences and Geophysical Fluid Dynamics Institute of Atmospheric Physics Chinese Academy of Sciences Beijing PR China; ^4^ Department of Environmental Sciences College of Agriculture and Environmental Sciences Government College University Faisalabad Faisalabad Pakistan; ^5^ Collaborative Innovation Center on Forecast and Evaluation of Meteorological Disasters Nanjing University of Information Science and Technology Nanjing PR China; ^6^ College of Hydrology and Water Resources Hohai University Nanjing PR China; ^7^ Institute of Geology University of Azad Jammu and Kashmir Muzaffarabad Pakistan

**Keywords:** population exposure, extreme event, trend analysis, Agroecological zones, Pakistan

## Abstract

The increasing prevalence of warmer trends and climate extremes exacerbate the population's exposure to urban settlements. This work investigated population exposure changes to mean and extreme climate events in different Agro‐Ecological Zones (AEZs) of Pakistan and associated mechanisms (1979−2020). Spatiotemporal trends in mean and extreme temperatures revealed significant warming mainly over northern, northeastern, and southern AEZs. In contrast, mean‐to‐extreme precipitation changes showed non‐uniform patterns with a significant increase in the northeast AEZs. Population exposure to mean (extreme) temperature and precipitation events increased two‐fold during 2000–2020. The AEZs in urban settlements (i.e., Indus Delta, Northern Irrigated Plain, and Barani/Rainfall) show a maximum exposure to extreme temperatures of about 70–100 × 10^6^ (person‐days) in the reference period (1979−1999), which increases to 140–200 × 10^6^ person‐days in the recent period (2000−2020). In addition, the highest exposure to extreme precipitation days also increases to 40–200 × 10^6^ person‐days during 2000–2020 than 1979−1999 (20–100 × 10^6^) person‐days. Relative changes in exposure are large (60%–90%) for the AEZs across northeast Pakistan, justifying the spatial population patterns over these zones. Overall, the observed changes in exposure are primarily attributed to the climate effect (50%) over most AEZs except Northern Irrigated Plain for R10 and R20 events, where the interaction effect takes the lead. The population exposure rapidly increased over major AEZs of Pakistan, which could be more vulnerable to extreme events due to rapid urbanization and population growth in the near future.

## Key Points

- Spatiotemporal trends in mean to extreme temperature (precipitation) events reveal widespread warming across Agro‐Ecological Zones
- Population exposure to mean (extreme) temperature and precipitation events increases two‐fold in recent climate period
- Observed changes in population exposure are primarily attributed to the climate effect (50%) over major Agro‐Ecological Zones

## 1 Introduction

The warming of the climate system is not uniform throughout the globe but varies in time and space (Alexander, 2016; Q. Sun et al., 2021). Over the last century, the estimated rate of global warming was about 1.1°C and is expected to rise by the end of the 21st century (Pörtner et al., 2022). The current Assessment Report (AR6) of the Intergovernmental Panel on Climate Change (IPCC) reported that the state of mean climate and its extremes are shifting toward a warmer climate (Legg, 2021). Exploring the likely changes in extremes requires further investigations beyond simply detecting a shift in the mean climate state (Mishra et al., 2015; Stocker, 2014). It is important to characterize changes in the tails of the distribution of climatic variables to thoroughly investigate their impacts on the environment and society (AghaKouchak et al., 2012; Lewis & King, 2017; Sillmann et al., 2017). Robust statistical analyses and reliable predictions are needed to detect and attribute changes in extremes and the risks associated with extreme events (Lavell et al., 2012; Seneviratne et al., 2012). The less‐developed regions are more vulnerable to the risks of extreme events. The ability to cope with significant weather and climate extremes impacts is solemnly dependent on sustainable policies and actions (Field & Barros, 2014).

In recent decades, several observed cases of extreme weather events infer a shift in the intensity and frequency of climate change extremes (Alexander, 2016; Beig et al., 2020; Singh et al., 2022; W. Zhang & Zhou, 2019; W. Zhang et al., 2019b, X. Zhang et al., 2023). With an increasing prevalence of warmer trends in the mean climate state, extreme weather events have become more frequent under a changing climate (Suarez‐Gutierrez et al., 2020; W. Zhang & Zhou, 2019; X. Zhang et al., 2011). Recurrent spells of heatwaves have become warmer than before and long‐lasting (Steffen et al., 2014), while extreme rainfall events (i.e., floods) become more recurrent and intense (Lee et al., 2018), which affect millions of people around the globe (Byers et al., 2018). The mid‐latitude land areas, including the Eastern United States, China, Southern Brazil, and Argentina, experienced extreme high wet‐bulb temperatures (Sherwood & Huber, 2010), where the earliest exposure to population making those regions more susceptible to climate risks (Byers et al., 2018; Kumar & Mishra, 2020; W. Zhang & Zhou, 2020). The 2019 extreme rainfall over East Africa was one of the wettest seasons (i.e., October–December) on record, causing flash floods, landslides and has affected around 2.8 million people (Wainwright et al., 2021). The frequency of extreme precipitation events has shown a persistent increase in rainy days in several regions across the US (Melillo et al., 2014), while the projected trends in the population exposure to extreme climate events are likely to increase (double) in the US by 2050 (Batibeniz et al., 2020). Varying patterns of climate change indicated a shift toward a warmer climate and more persistent extreme events worldwide (Alexander, 2016; Byers et al., 2018; Kumar & Mishra, 2020; Sillmann et al., 2017; Q. Sun et al., 2021).

Extreme climate events have significantly impacted Pakistan’s socioeconomic and environmental conditions (Abbas et al., 2018a; Adnan et al., 2016; Hina et al., 2021; Javaid & Chawla, 2019). The spatiotemporal patterns of extreme minimum (maximum) temperatures have shown significant warmer trends in observed records for Pakistan (Hina & Saleem, 2019; A. Hussain et al., 2023a; M. A. Iqbal et al., 2016; Zahid & Rasul, 2012). The Indus Delta of Sindh and Punjab province experienced an increase in summer day’s temperatures (Abbas, 2013; Abbas et al., 2018a; Nawaz et al., 2019). Trends in extreme heat spells led to widespread warming across Pakistan and impacted more than 50% of the total population (N. Khan et al., 2019a). Largescale changes in atmospheric fields were responsible for the occurrence of extremely hot days in the region which could further lead to severe heatwave conditions in the presence of a low‐pressure system (N. Khan et al., 2019b). Precipitation regime varies dramatically in time and space, indicating an abrupt climate change in the region (Beck et al., 2015; A. Hussain et al., 2022b, 2023b). A linearly increasing trend has been apparent for the annual total rainfall events in north‐western Pakistan (Ahmed et al., 2017; Gadiwala & Burke, 2019; A. Hussain et al., 2022a) whereas a decreasing trend was noticed toward the southern parts (Hanif et al., 2013; M. S. Hussain & Lee, 2014). The inter‐annual to seasonal changes in mean and extreme precipitation events over Pakistan are also evident in literature (Asmat & Athar, 2017; Hartmann & Buchanan, 2014; M. S. Hussain & Lee, 2014; Sheikh et al., 2015). The heavy rainfall event of the 2010 Pakistan flood was strongly connected to largescale atmospheric Rossby wave forcing (Lau & Kim, 2012) which has affected around 20 million people in the region and displaced approximately 11 million in numbers (Arif et al., 2019). The persistent increase in extreme weather and climate events, associated largescale atmospheric patterns, and societal impacts in Pakistan are critical to analyze for mitigation and adaptation measures.

The population of Pakistan is experiencing rapid growth and is projected to double by 2050 (DESA, 2019). Nevertheless, the extent of exposure of the region’s population to weather and climate extremes remains largely unknown. The present study sought to quantify population exposure changes to mean and extreme climate events in observed records (1979−2020) across AEZs of Pakistan. This study is the first of its kind for the region because none of the studies mentioned above has calculated human exposure to climate extremes in AEZs. We aim to address the following research questions: (a) What are the characteristics of extreme climate over Pakistan and related largescale mechanisms? (b) What are the associated impacts on population in observed records? and (c) What are the relative roles of changes in climate and population on the impacts? This kind of research is critical for the institutional and policy landscape to revisit climate change adaptation plans and provide effective climate services to mitigate climate risks and impacts.

## 2 Materials and Methods

### 2.1 Study Area

**Figure 1:**
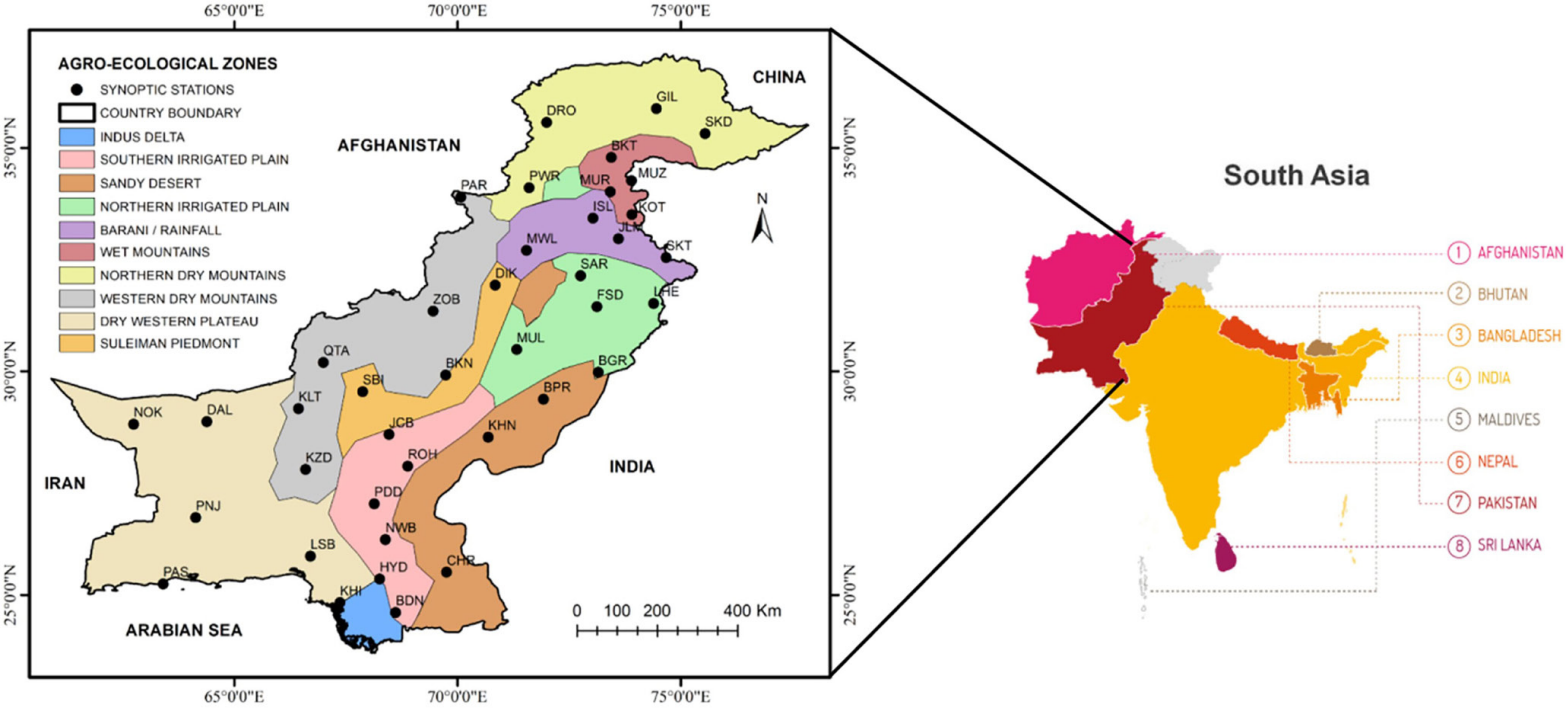
Map of South Asia with a focus on Pakistan, showing Agro‐Ecological Zones and spatial distribution of weather observatories.

Pakistan, with an area of 796,096 km^2^, is located in South Asia and is positioned between latitudes 23°N–37°N and longitudes 60°E–76°E. The country’s geography is diverse and intricate, stretching from the Himalayas and Karakoram ranges in the northwestern region to the fertile plains of the Indus River basin and the Arabian Sea in the southern part (Hina & Saleem, 2019). The elevation of Pakistan ranges between 0 and 8,611 m above sea level in the North. According to the climatic classification, 70%–88% of the country has an arid to semiarid climate (Adnan et al., 2018). The highest mean temperatures (above 35°C) are recorded in the central and southeastern parts of the country (Asmat & Athar, 2017; M. F. Iqbal & Athar, 2018). The hydrological cycle in Pakistan varies across different latitudinal belts. The lower regions experience minimal precipitation (less than 150 mm) annually, while the upper regions receive higher amounts (over 500 mm) (Bhatti et al., 2020). Based on the geography, climate, and agriculture, the country (Figure 1) has been classified into 10 AEZs. A detailed description of these AEZs is given in PARC (1980) and Saleem et al. (2021). The topographic and climatological features of various AEZs are also provided in Table S1 of the Supporting Information S1.

### 2.2 Data Collection

The data sets containing daily minimum (*T*_min_), maximum (*T*_max_) temperatures, and daily precipitation (Prcp) were obtained from the Pakistan Meteorological Department for the period spanning 1979−2020. Data quality control and homogenization are critical for trend analysis studies (Abatan et al., 2016). Hence, the weather stations with available long‐term records (i.e., less than 5% data is missing) and have spatial coherence to various AEZs are chosen for further data processing and analyses. The detailed descriptions of selected meteorological stations are given in (Table S1 in Supporting Information S1). The use of gridded climate products in complex terrain produces uncertainties in results, which may affect the long‐term trend of climate variables (Baudouin et al., 2020; Dahri et al., 2021; Nawaz et al., 2020). Therefore, in this study, we make use of high‐quality observational data with sufficient spatial and temporal coverage across AEZs of Pakistan. We also evaluated the data quality using autocorrelation and pre‐whitening tests (Figure S1 in Supporting Information S1). This work does not account the intricacies of meso and microscale meteorology, influence of topography, and specific localized factors (i.e., urban heat island effect and land use). Consequently, any conclusions drawn should be viewed with this constraint in mind.

In order to study large scale drivers of climate variability and change in the region, the ERA5 monthly reanalysis data (i.e., 0.25° grid) of single and multi‐level variables are used (1979–2020). To calculate population exposure to extreme climate indices, the annual population data from the Inter‐Sectoral Impact Model Intercomparison Project (ISIMIP) protocol ISIMIP2b (https://www.isimip.org) are used. The ISIMIP historical population data are available till 2005; onwards, the projected population scenario of SSP2 (Shared Socioeconomic Pathways) as the middle of the road was used (2006−2020) to calculate exposure.

### 2.3 Trend Analysis

Temperature and rainfall indices are calculated on an annual timescale using RClimDex (v1.9) software (X. Zhang et al., 2018) with reference to a base period of 1981–2010 (Table 1). The extreme climate indices are examined to detect trends and their magnitude using a nonparametric Mann Kendall (MK) test (Kendall, 1955; Mann, 1945) and Sen’s slope estimator method (Sen, 1968). The degree of lag−1 autocorrelation was first tested on Tmin, Tmax and Prcp time series before applying MK and SS tests. Prewhitening test is used to eliminate the effect of serial correlation in time series data (Figure S1 in Supporting Information S1). When the time series of indices did not depict a significant autocorrelation, the original MK test was applied to detect the trend. Subsequently, the trend values for the individual stations within a specific zone, as well as for the entire country of Pakistan, were averaged separately to compute zonal trends (Table 2). A confidence interval of 95% was selected to determine the significance of each trend. Moreover, the anomalous patterns of low‐level cloud cover, surface thermal and solar radiations, zonal winds at 200 and 850 hPa, relative divergence at 200 hPa, vertical velocity at 200 and 850 hPa, vertical integrated moisture transport (Latif et al., 2017, 2018) from 850 to 300 hPa, and moisture flux convergence/divergence (K. Ullah & Gao, 2012) are presented to study large scale atmospheric dynamics in the region. Student’s *t*‐test is used to calculate trends significance at a 95% level. Monthly anomalies are derived by subtracting the climatological monthly means from the corresponding data.

**Table 1:** List of Core Extreme Climate Indices Recommended by the Experts Team on Climate Change Detection and Indices (X. Zhang et al., 2018)

	Indices	Name	Definition	Units
Temperature	T_MEAN_	Average temperature	Mean of maximum (TX) and minimum (TN) temperature	°C
	TNx	Hottest nights	Monthly maximum value of daily minimum temperature	°C
	TX90p	Warm days	Percentage of days when TX > 90th percentile	days
Precipitation	P_MEAN_	Average precipitation	Mean of the annual total precipitation (rainfall ≥ 1 mm)	mm
	R10	Heavy precipitation days	Annual count of days when PRCPTOT ≥ 10 mm	days
	R20	Extreme precipitation days	Annual count of days when PRCPTOT ≥ 20 mm	days

**Table 2:** Annual Trend (Decade^−1^) of Extreme Temperature and Precipitation Indices Across Different Zones of Pakistan

	Indices	Agro‐ecological zones (AEZs)									
		Zone^1^	Zone^2^	Zone^3^	Zone^4^	Zone^5^	Zone^6^	Zone^7^	Zone^8^	Zone^9^	Zone^10^
Temperature	T_MEAN_	0.33*	0.44*	0.31*	0.40*	0.30*	−0.29*	−0.22	0.21*	0.30*	0.22*
	TNx	0.53*	0.51*	0.32*	0.60*	0.35*	−0.23	−0.20	0.13	0.35*	0.41*
	TX90p	1.10	1.40	1.44	1.25*	1.00*	−3.05	−1.55	1.13	2.43*	1.33*
Precipitation	P_MEAN_	−0.40	0.27	0.20	0.46*	0.38	0.82*	0.50	−0.44	−0.58	0.62
	R10	−0.26	0.16	0.27	0.21	0.00	1.63*	0.61	−0.37	−0.41	0.50
	R20	−0.20	0.15	0.11	0.00	0.33*	0.51	0.37*	−0.23	−0.14	0.23

*Note*. Zones description: Indus Delta^1^; Southern Irrigated Plain^2^; Sandy Desert^3^; Northern Irrigated Plain^4^; Barani/Rainfall^5^; Wet Mountains^6^; Northern Dry Mountains^7^; Western Dry Mountains^8^; Dry Western Plateau^9^ and Suleiman Piedmont^10^, respectively. Significance (*) of trends at 95% level was calculated using Mann‐Kendall's test.

### 2.4 Exposure to Extreme Events

To quantify human exposure to mean and extreme climate events, the ISIMIP population data is used, with a horizontal resolution of 0.5° (∼50 km). In order to calculate exposure on a consistent spatial unit, the extreme events data at station level are interpolated to a regular grid of 0.5° using iterative improvement objective analysis (https://www.ncl.ucar.edu/Document/Functions/Built-in/obj_anal_ic.shtml). Afterward, population exposure is estimated at each grid point, and regional averaged exposure is calculated in each zone. Notably, we divide the data sets into two twenty‐one (21) years periods. The first 21 years period is termed as “reference period” (1979−1999), and the next 21 years are referred to as “recent period” (2000−2020). Later in each grid cell, we calculate the exposure as a product of the population and the count of mean and extreme climate events for both the reference and recent time periods. The exposure units of population are given in person‐days (Figure 4). To assess the relative importance of various drivers of change in exposure, we employ a methodology developed by Jones et al. (2015). The change in exposure (ΔE) is quantified by summing the effects of climate, population, and their interaction as follows:

(1) $$∆E=P_{R}∆C+C_{R}∆P+∆C∆P$$

where Δ*E* indicate the total changes in population exposure, *P*_*R*_ and *C*_*R*_ represent the population and climate in the reference period. Whereas Δ*C* and Δ*P* signify the changes in climate and population in the recent period. The *P*_*R*_Δ*C* explains the climate effect, which takes into account the influence of climate change, the *C*_*R*_Δ*P* defines the population effect, and the Δ*C*Δ*P* reflects the combined (interaction) effects of simultaneous change in climate and population. To determine the percentage change associated with each effect, we divide the aforementioned equation by the exposure observed during the reference period.

## 3 Results

### 3.1 Temperature Changes and Mechanisms

**Figure 2:**
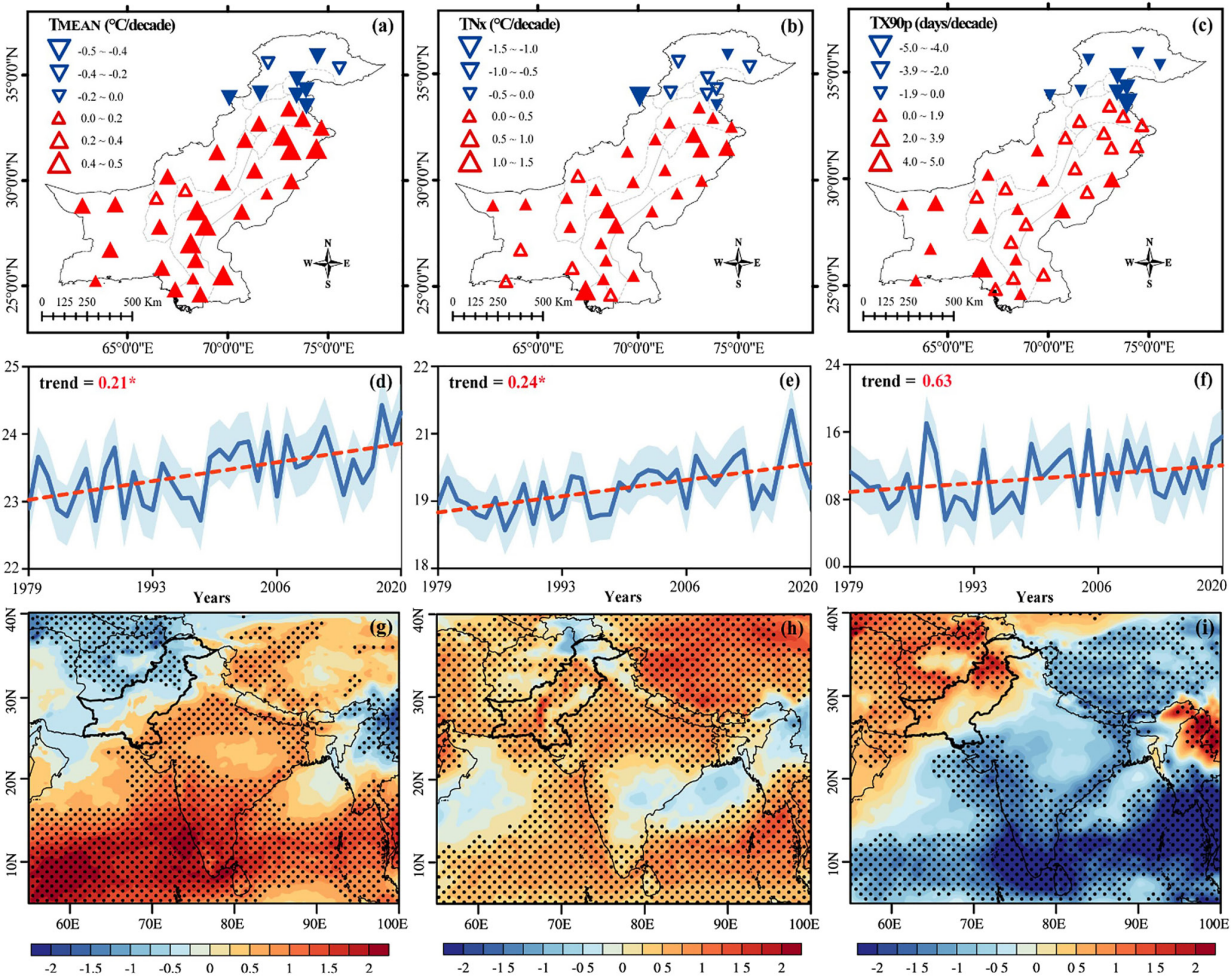
Spatial patterns and trends (decade^−1^) of extreme temperature indices during 1979–2020. The panels are for the (a, d) T_MEAN_; (b, e) TNx; (c, f) and TX90p. Upward (downward) pointed triangles indicate increasing (decreasing) trends. Filled triangles indicate significant trends at the 95% confidence level. The size of the triangle indicates trends magnitude (shown in the legend). The shaded light‐blue area shows deviation from the mean (solid dark‐blue line). The slope inter‐decadal rates are given on each panel plot highlighted with red color, where * represents significant change at 95% confidence level (d, e, f). Trends of anomalous (g) cloud cover (%), (h) surface thermal radiation (W/m^2^), and (i) surface solar radiation (W/m^2^). The black dots highlight the areas significant at 95% level, calculated using Student's *t*‐test.

The spatiotemporal trends in annual mean and extremes temperature events are given in Figure 2. Overall, an increasing trend in *T*_MEAN_ is evident during the analysis (1979−2020) period of past 42−years. On an annual timescale, 31 out of 40 stations reveal warmer (increasing) trends for *T*_MEAN_ temperature; however, the estimated percentage of significant increasing (decreasing) trends is 72.5% (17.5%), respectively (Figure 2a). The regional average long‐term trend of *T*_MEAN_ is significant, 0.21°C decade^−1^ (Figure 2d), indicating a warmer climate for the region. While discussing the zone wise trends of *T*_MEAN_, almost all zones exhibited significant increasing trends ranging from 0.21 to 0.44°C decade^−1^ (Table 2), except Wet Mountains (−0.22°C decade^−1^) and Northern Dry Mountains (−0.29°C decade^−1^). These AEZ’s are identified as the hotspot regions in Pakistan and are most vulnerable to warming (Adnan et al., 2017).

A significant increasing trend has been observed in the region for the TNx index (0.24°C decade^−1^) since 1979 (Figure 2e). Among 40 stations, 65% (26) of the stations witnessed significant warming trends for TNx (Figure 2b). The analysis of trends (decade^−1^) in TNx temperature exhibited robust warming in most of the AEZs having arid to semiarid climatic conditions (Table 2). The percentage of stations with significant positive (negative) trends in TX90p is counted as 35% (17.5%), depicting a hotter climate in observed records (Figure 2c). The per decade trend in TX90p index (Table 2) has shown an increasing pattern with a significant rise in Northern Irrigated Plains (1.25 days decade^−1^), Barani/Rainfall (1.00 days decade^−1^), Dry Western Plateau (2.43 days decade^−1^) and Suleiman Piedmont (1.33 days decade^−1^). These findings align with previous studies in the literature (Abbas, 2013; Nawaz et al., 2019). However, there are slight discrepancies between their results and those of this study, which may be attributed to variations in the study period or statistical methods employed.

The possible mechanisms responsible for warming in the region are low‐level cloud cover (Figure 2g), changes in surface thermal radiation flux (Figure 2h), and surface solar radiation flux (Figure 2i), respectively. The high pressure over Northeastern Pakistan can weaken the South Asian subtropical upper‐level westerly jet (Figure S2a in Supporting Information S1), causing a significant upper‐level convergence over Pakistan (Figure S2b in Supporting Information S1). Due to the convergence of upper‐level airflow over Pakistan, the mass continuity produces a compensating downward flow. Consequently, a significantly sinking motion trend can be observed for the region (Figure S2c in Supporting Information S1), which can further reduce low‐level clouds (Figure 2g). Furthermore, the reduced cloud cover favors downwards shortwave radiation (Figure 2i), which upsurges the land surface temperature. Besides this, the significant warming trends of surface thermal radiation at 18:00 UTC (11:00 p.m. local time) from the land surface may further warm the overlying atmosphere over Pakistan (Figure 2h).

### 3.2 Precipitation Changes and Mechanisms

**Figure 3:**
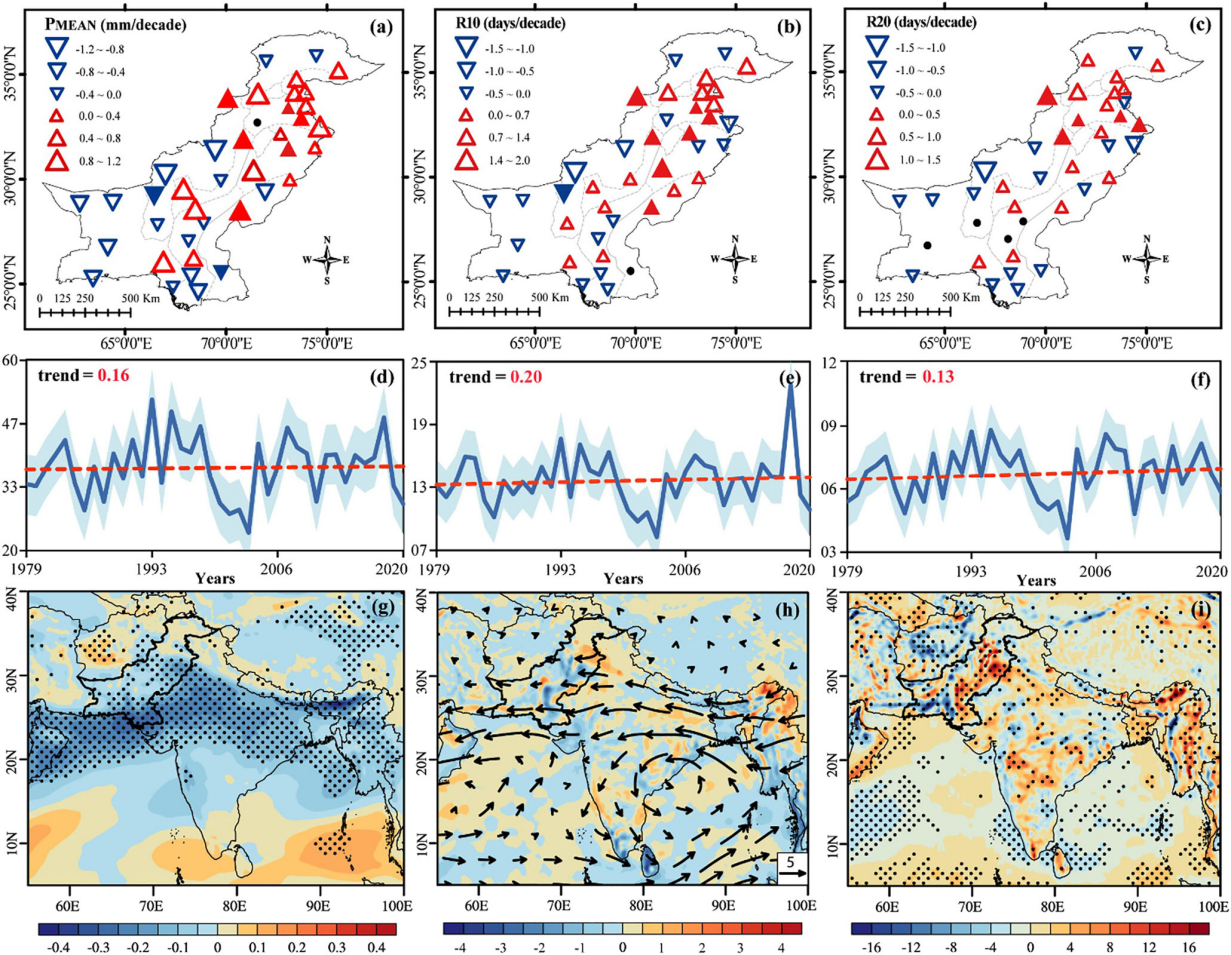
Spatial patterns and trends (decade^−1^) of extreme precipitation indices during 1979–2020. The panels are for the (a, d) *P*_MEAN_; (b, e) R10; (c, f) and R20. Upward (downward) pointed triangles indicate increasing (decreasing) trends. Filled triangles indicate significant trends at the 95% confidence level. The size of the triangle indicates trends magnitude (shown in the legend). The shaded light‐blue area shows deviation from the mean (solid dark‐blue line). The slope inter‐decadal rates are given on each panel plot highlighted with red color, where * represents significant change at 95% confidence level (d, e, f). Trends of anomalous (g) 850 hPa zonal wind (m s^−1^), (h) vertically integrated moisture flux (shaded; 10^−6^ kg m^−2^ s^−1^) and transport (vector; kg m^−1^ s^−1^) from 850 to 300 hPa, and (i) vertical velocity (10^−3^ Pa s^−1^) at 850 hPa, respectively. The black dots highlight the areas significant at 95% level, calculated using Student's *t*‐test.

Figure 3 shows the spatiotemporal trends in annual mean and extremes precipitation events during 1979–2020. Spatial trends in mean precipitation (*P*_MEAN_) events show non‐uniform patterns with large spatial differences (Figure 3a). The percentages of stations depicting positive (negative) trends in *P*_MEAN_ are 52% (45%), with significant trends of about 15% (5%). On an annual timescale, a slightly increasing and non‐significant trend (0.16 mm/decade^−1^) is noticed for *P*_MEAN_ (Figure 3d). Zone‐wise analysis further reveals a general increase in *P*_MEAN_ events in all AEZs except Indus Delta, Western Dry Mountains and Dry Western Plateau (Table 2). The zones of Northern Irrigated Plain (0.46 mm/decade^−1^) and Wet Mountains (0.82 mm/decade^−1^) show statistically significant positive trends in *P*_MEAN_. The year‐to‐year rainfall variability could be affected by the altitude of stations within these AEZs (Abbas et al., 2014).

The extreme R10 (R20) precipitation events has a similar spatial pattern like *P*_MEAN_, but show larger trend values (Figures 3b and 3c). The proportion of stations showing positive trends in R10 (R20) is 52% (50%); among them, only 17% (12%) of the stations reveal significant changes. The regional averaged long‐term trend of R10 (R20) precipitation events is calculated to be 0.20 (0.13) days decade^−1^ since 1979 (Figures 3e and 3f). The zone of Wet Mountain is the only AEZs showing a statistically significant trend for R10 precipitation events. While, in the case of R20, the two AEZs (zone^5^ and zone^7^) shows statistically significant trends (Table 2). Overall, the increasing trends are apparent for the extreme precipitation events across some of the central and northeastern AEZs, while southwestern arid to semiarid zones showed opposite patterns during 1979–2020.

Spatial patterns of zonal wind at 850 hPa reveal a trough like structure across southern and central Pakistan (Figure 3g). The easterly anomalies in the lower‐troposphere favor moisture transport, originating from the Bay of Bengal and, to some extent, from the Arabian Sea (Figure 3h). This pattern of low‐troposphere convergence results in compensating upward vertical motion across the latitudes between 24 and 34°N. Subsequently, there are significant local ascending motions (i.e., forming clouds and precipitation in the region) over 24–34°N, which corresponds to higher values of mean and extreme precipitation events (Figure 3i).

### 3.3 Population Exposure to Extreme Climate Events

**Figure 4:**
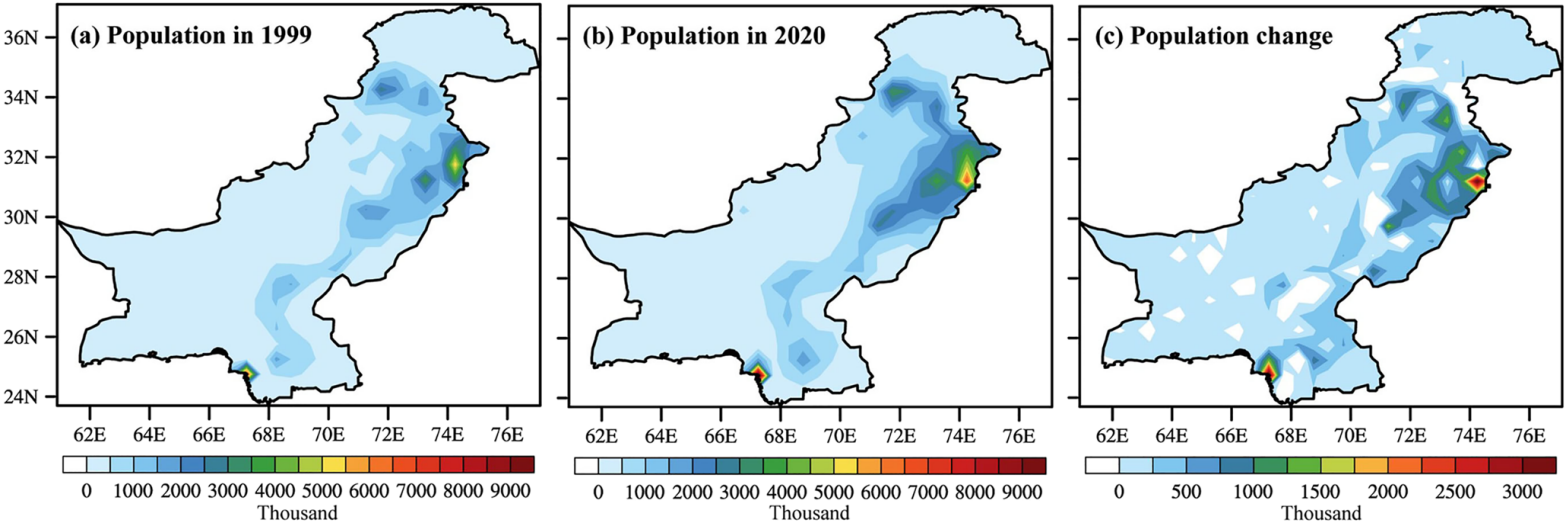
Spatial distribution of population (units: thousand persons) during 1999 and 2020. Changes in population redistribution are calculated with reference to base year of 1999.

Spatial patterns and changes in population redistribution are given in Figure 4. A two‐fold increase in the population density is noticed, mainly in the northeast, central and southeastern Pakistan (Figures 4a and 4b) and changes in population redistribution further confirm this pattern (Figure 4c).

**Figure 5:**
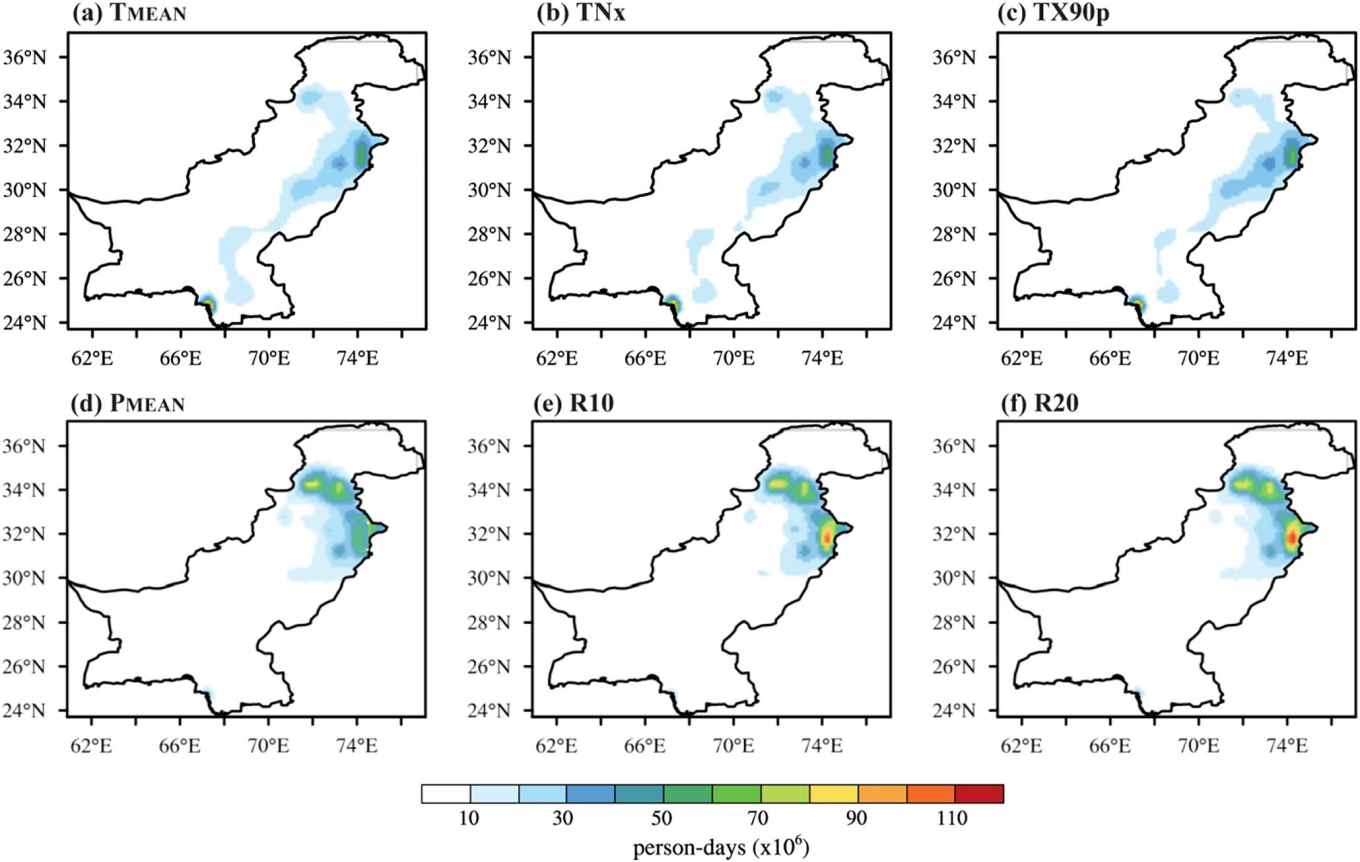
Population exposure to extreme climate events for the reference period (1979−1999).

Population exposure to mean (Figure 5a) and extreme (Figures 5b and 5c) temperature events are found to be 1–10 × 10^6^ person‐days during 1979–1999. While, zones of Indus Delta, Southern Irrigated Plain, Northern Irrigated Plain, and Barani/Rainfall experienced the maximum exposure ranging from 20 to 60 × 10^6^ person‐days. The highest population exposure to mean precipitation is between 20 and 70 × 10^6^ person‐days (Figure 5d). The AEZs largely exposed to *P*_MEAN_ are Northern Irrigated Plain, Barani/Rainfall, and Wet Mountains. We further quantify the population exposed to extreme rain on more than 10 (20) days on an annual timescale because the impact on people varies depending on whether they are exposed to such events. The zones with highest (lowest) population exposure to mean (Figure 5d) and extreme (Figures 5e and 5f) precipitation events are located in northeast (southwest) Pakistan. The magnitude of exposure to extreme precipitation events is larger than mean rainfall, respectively.

**Figure 6:**
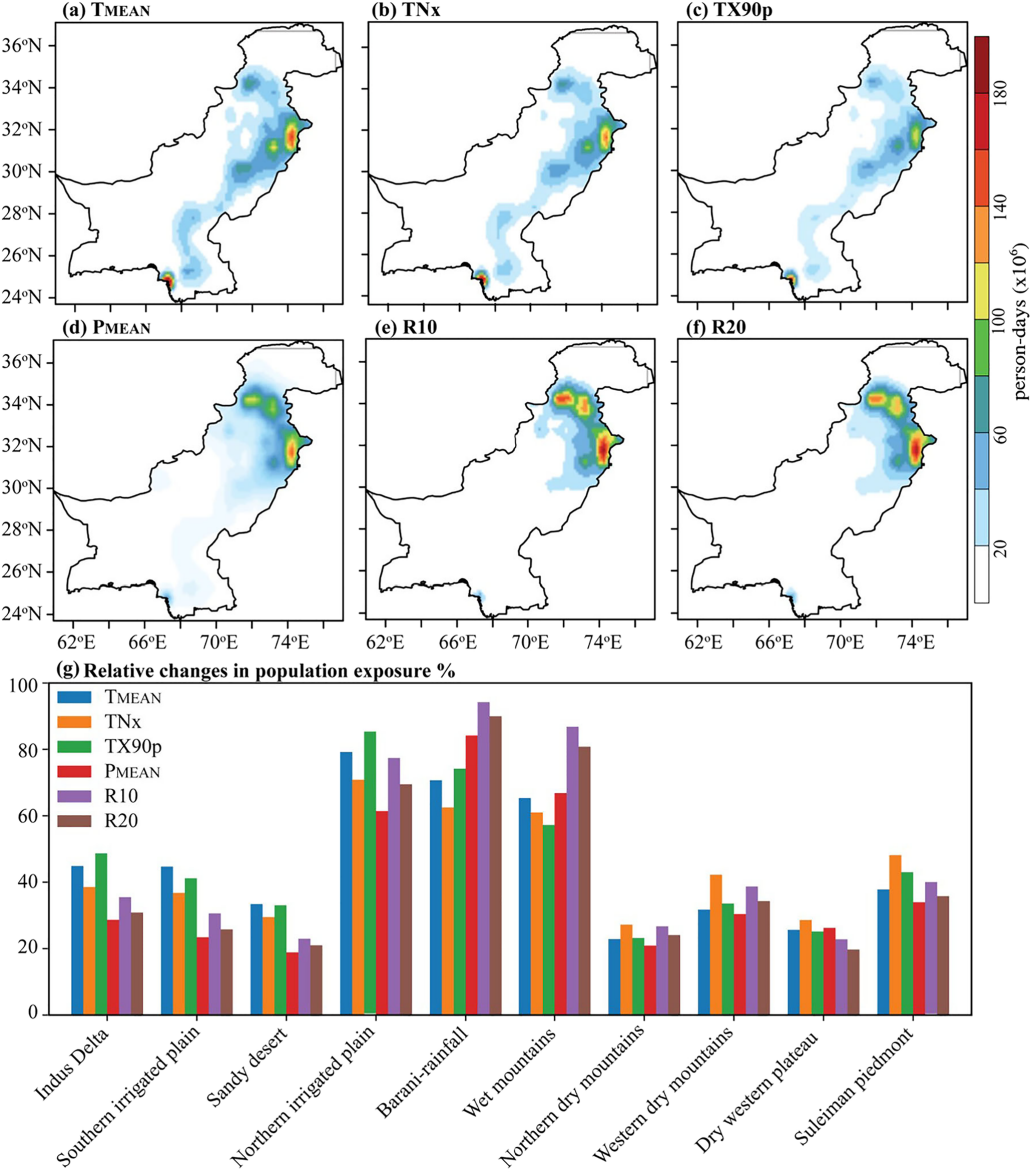
Population exposure to temperature (a–c) and precipitation (d–f) events during (2000−2020) recent climate period. The bar plots (g) indicate relative changes in population exposure (%) across different zones of Pakistan. Relative changes are calculated with reference to base period (1979−1999).

Figure 6 shows the spatial distribution of exposed population and relative changes in the recent period (2000−2020). Population exposure to temperature (Figures 6a−6c) and precipitation (Figures 6d−6f) extremes depict similar patterns as seen in the reference period but show greater magnitude and areal extent during 2000–2020. The increase in population exposure to *T*_MEAN_ is largest (i.e., ranges between 40 and 160 × 10^6^ person‐days) for the zones of Indus Delta, Southern Irrigated Plain, Northern Irrigated, Barani/Rainfall and Wet Mountains, with a two‐fold increase in exposure relative to reference period (Figure 6a). A large portion of AEZs have been exposed to TNx (TX90p) events in the recent period, with a magnitude varying between 40 and 150 × 10^6^ person‐days (Figures 6b and 6c). The annual maximum exposure to *P*_MEAN_ in the reference period is (20 − 70 × 10^6^ person‐days), which has increased to (50 − 150 × 10^6^ person‐days) in the recent period (Figure 6d). Similarly, the highest exposure to extreme R10 (R20) rainy days (Figures 6e and 6f) also increases to 40 − 180 × 10^6^ person‐days (whereas the exposure in the reference period is 20–110 × 10^6^ person‐days). Overall, a two‐fold increase in exposure to extreme temperature (precipitation) events is evident during 2000–2020, while some AEZs (i.e., Northern Irrigated Plain, Barani/Rainfall, and Wet Mountains) even show larger values in population exposure.

Figure 6g shows the relative changes in population exposure (%) to extreme climate events across AEZs of Pakistan. Relative changes are calculated with respect to the reference period. On the annual timeframe, population exposure increases the most, 60−90% for the zones of Northern Irrigated Plain, Barani/Rainfall, and Wet Mountains and least, 20%−30% for Sandy Desert, Northern Dry Mountains, and Dry Western Plateau. Notably, the annual aggregated exposure is high for the AEZs across northeast Pakistan, justifying the spatial population exposure patterns over these zones. In addition, zones of Indus Delta, Southern Irrigated Plains, and Suleiman Piedmont revealed slight changes in population exposure (30%−40%) relative to the reference period. Overall, it can be inferred from the results that the population exposure rapidly increased over major AEZs of Pakistan.

### 3.4 Decomposition of Changes in Exposure

**Figure 7:**
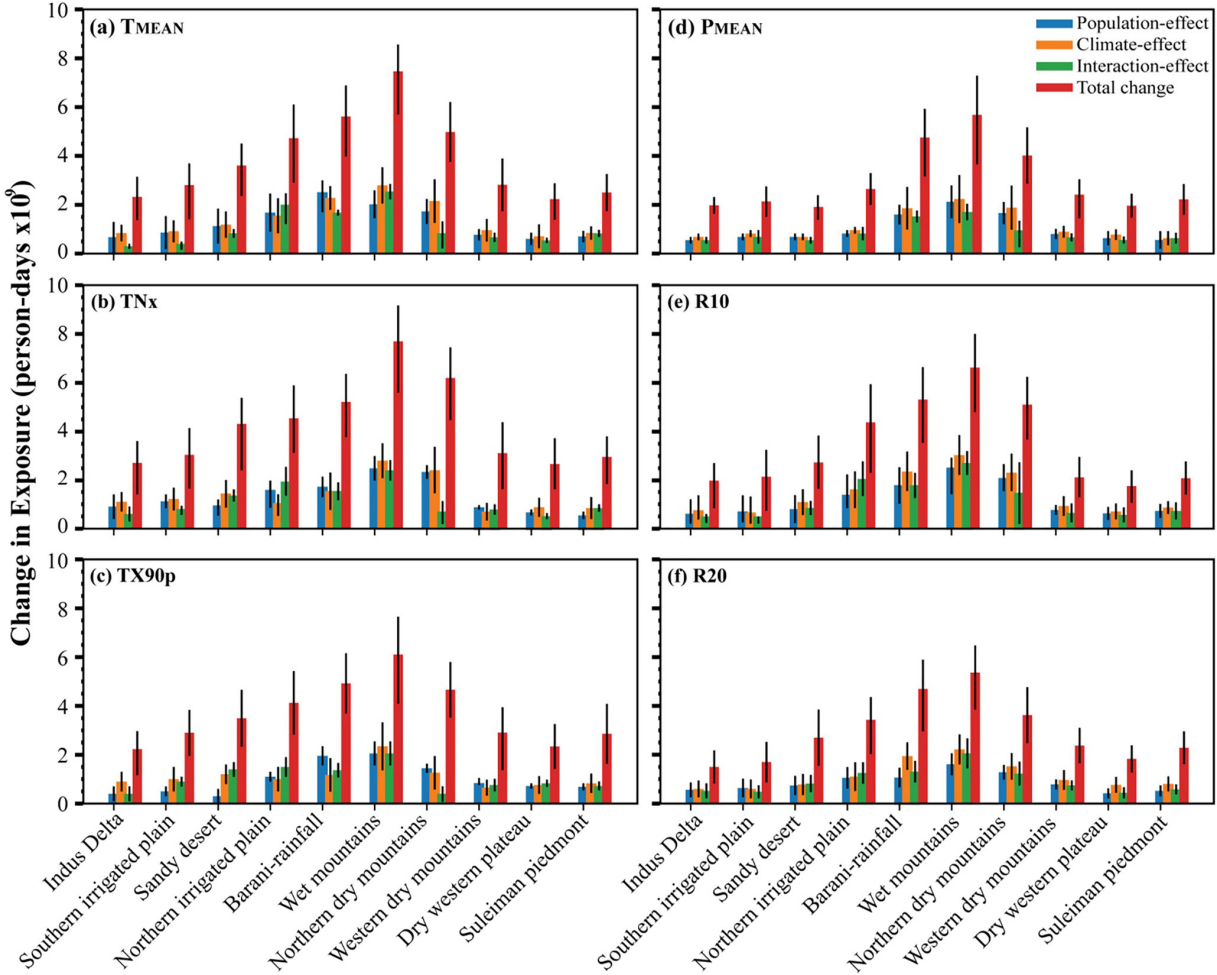
Decomposition of exposure changes into population, climate, interaction and total effect over various zones of Pakistan. Error bars indicate the deviation from the mean values.

To investigate the relative importance of various factors (drivers), we decomposed the population exposure into climatic, population, interaction and total effects (see Section 2 for details). Figure 7 shows changes in exposure and its components across various AEZs of Pakistan. For extreme temperature (*T*_MEAN_, TNx, TX90p) events, the effect of climate accounts nearly half of the total change in population exposure (40%–50%) in most AEZs, followed by population effect (20%–30%) and the interaction effect (10%–20%), respectively. A higher magnitude of total change in exposure is noticed (6 × 10^9^–8 × 10^9^ person‐days) for the zone of Northern Irrigated Plain, Barani/Rainfall, Wet Mountains and Northern Dry Mountains. Notably, the Northern Irrigated Plain is the only AEZs that depict a more pronounced (larger) interactive effect than climate and population for *T*_MEAN_ (Figure 7a), TNx (Figure 7b) and TX90p (Figure 7c) events. The observed zone‐wise changes in exposure to *P*_MEAN_, R10, and R20 events (Figures 7d−7f), depict similar patterns to temperature extremes but show relatively smaller effects. Changes in exposure to precipitation events are primarily attributed to the climate effect (50%) over most AEZs except in the Northern Irrigated Plain for R10 (R20) events, where the interaction effect 50% (40%) takes the lead. In general, the AEZs (i.e., Barani/Rainfall, Wet Mountains, and Northern Dry Mountains) indicate larger change in exposure to *P*_MEAN_, R10, and R20 events. In addition, the slight magnitude changes in total exposure are associated mainly with fewer populations in those AEZs across the region.

## 4 Discussion

The significant impacts of extreme weather and climate events on society are evident in literature (Pour et al., 2014; Salman et al., 2017; Shahid et al., 2017). With the increasing prevalence of warmer trends, the risk of population exposure to extreme events increases in the climatological stressed regions (Dutta & Chaudhuri, 2015; Nashwan et al., 2019; Salguero‐Gomez et al., 2012). In the past decade, many scientists investigated climate variability induced temperature (precipitation) patterns and extreme weather events in Pakistan (Abbas et al., 2018a, 2018b; Asmat & Athar, 2017; Asmat et al., 2018; Bhatti et al., 2020; Saleem et al., 2021). While, it is largely unknown how exposed the region’s population is to extreme climate events.

Most of the AEZs in Pakistan reveals widespread warming trends in mean (Figure 2a) and extreme temperature events (Figures 2b and 2c). Whereas, fewer AEZs in the northern Pakistan show cooling trends, which might be due to direct (indirect) radiative forcing of anthropogenic aerosols (Z. Wang et al., 2016). Overall, a significant warmer trend is observed in the region for *T*_MEAN_ (0.21°C decade^−1^) and TNx (0.24°C decade^−1^) since 1979 (Figures 2d and 2e). The calculated percentage of significant warming trends in *T*_MEAN_, TNx and TX90p events are 75%, 65%, and 35% (Figures 2a−2c), depicting an increase in the frequency and intensity of temperature extremes across AEZs in southern and central Pakistan. Previous work on changes in mean and extreme temperatures also revealed significant warming trends (X.‐B. Sun et al., 2017; You et al., 2017). However, there are slight discrepancies between their results and those of this study, which may be attributed to variations in the study period or statistical methods employed. The zone‐wise analysis of extreme temperature indices across various AEZs of Pakistan revealed warming (1979−2020), having arid to semiarid climate conditions (Table 2). The hotter or drier weather exerts a lot of pressure on water requirements for agricultural and socioeconomic sectors within these AEZs (Adnan et al., 2017). Analysis of large‐scale circulation patterns revealed that low‐level cloud cover and changes between land‐atmosphere radiational feedback processes are the likely cause of extreme‐high temperatures in the region (Figures 2g−2i). Previous studies on atmospheric circulation further confirm these potential mechanisms (Hong et al., 2020).

Spatial trends in mean (Figure 3a) to extreme precipitation (Figures 3b and 3c) events show non‐uniform behavior with large spatial differences. The elevation of stations in AEZs may influence year‐to‐year rainfall variability (Abbas et al., 2014). The percentage of stations with increasing trends in *P*_MEAN_ R10 and R20 events are 52%, 52%, and 50%. Whereas, the long‐term trend is found to be insignificant (Figures 2d−2f). The annual averaged trends in mean to extreme precipitation events over different AEZs varies from one zone to another (Table 2). The AEZs in the northeast region generally experienced more extreme rainy days than the southwest. These AEZs are at a larger risk to climate change (Saleem et al., 2021). The proximity of weather stations to the westerly disturbance route or the core monsoon zone of Pakistan might be one of the factors for positive and significant trends in the heavy tails of precipitation (Asmat & Athar, 2017; Dimri et al., 2015; S. Y. Wang et al., 2011). The spatial trends in zonal winds at 850 hPa reveal a trough like structure over southern and central Pakistan (Figure 3g). The easterly anomalies in the lower troposphere reveal a strong link with Pakistan rainfall, depicting the significant role of zonal wind anomalies in affecting rainfall through water vapor transport from ocean to land (Lu et al., 2020).

A robust increase in the population density is evident over time, primarily in the urban areas across northeast, central and southeastern Pakistan (Figure 4). Population exposure to mean and extreme temperature events increase two‐folds during recent climate period of 2000–2020. The AEZs in urban settlements (i.e., Indus Delta, Southern Irrigated Plain, Northern Irrigated Plain, and Barani/Rainfall) show an exposure value between 20 and 60 × 10^6^ person‐days during 1979–1999 (Figures 5a−5c), which increases to 40 − 140 × 10^6^ person‐days during 2000–2020 (Figures 6a−6c). While, some AEZs even show larger exposure values during the reference (recent) period. Urban heat island is a key indicator that exacerbates the areal extent and magnitude of exposure to temperature extremes in urban settlements (Hajat & Kosatky, 2010; Li & Bou‐Zeid, 2013; Mishra et al., 2015; Thirumalai et al., 2017). Estimating uncertainties when calculating exposure to heat‐related extremes is vital, and caution should be taken when choosing different methods and data sets. Besides, our results agree with the findings of previous studies on exposure to heat extremes (Chen et al., 2020; Liu et al., 2020; I. Ullah et al., 2022a; W. Zhang & Zhou, 2020).

Population exposure to mean and extreme precipitation events are larger for 2000−2020 than 1979−1999, due to the rapid population growth in time and space combined with the increase in rainfall extremes over these zones (Table 2). The annual maximum *P*_MEAN_ exposure (Figure 5d) in the reference period is (20–70 × 10^6^ person‐days), which has increased to (50–150 × 10^6^ person‐days) in the recent period (Figure 6d). Similarly, the highest exposure (Figures 6e and 6f) to extreme R10 and R20 rainy days also increases to 40–180 × 10^6^ person‐days (whereas the exposure (Figures 5e and 5f) in the reference period is 20–110 × 10^6^ person‐days). Relative changes in exposure are large (60%–90%) for the AEZs across northeast Pakistan, justifying the spatial population exposure patterns over these zones (Figure 6g). Interannual rainfall variability, temperatures changes and land‐use patterns are the key factors, exacerbating population exposure changes over arid to semiarid regions (Hina et al., 2021; Saleem et al., 2021). Overall, it can be inferred from the results that the population exposure rapidly increased over major AEZs of Pakistan, which could be more vulnerable to extreme events due to rapid urbanization and population growth in the near future (I. Ullah et al., 2022b).

The relative importance of exposure into population, climate, interaction and total effect is given in Figure 7. For extreme temperature (*T*_MEAN_, TNx, TX90p) events, the effect of climate accounts nearly half of the total change in population exposure (40%–50%) in most AEZs. In contrast, the effect of population change (20%–30%) is greater than the interaction effect (10%–20%), respectively. Notably, the Northern Irrigated Plain is the only AEZs that depict a more pronounced (larger) interactive effect than climate and population (Figures 7a−7c). For extreme precipitation (*P*_MEAN_, R10, R20) events, the observed zone‐wise changes in exposure depict similar patterns as temperature extremes but show relatively smaller effects (Figures 7d−7f). Overall, the observed changes in exposure are primarily attributed to the climate effect (50%) over most AEZs except Northern Irrigated Plain (i.e., R10 and R20 events), where the interaction effect takes the lead. The increase in the influence of interactive effect reveals the importance of interactions between population and climate change in increasing total change in exposure. It is evident that a warmer climate is anticipated to increase the rainfall intensity over Asia and increases the risk of population exposure to these extreme events. The quantification of people exposed to extreme climate events is key to assessing the risks associated with temperature and precipitation extremes (Chen et al., 2020; Liu et al., 2020; W. Zhang & Zhou, 2020; P. Zhang et al., 2019a).

It is important to look at temperature and precipitation variability in AEZs because warming is not homogeneous across the region and different socioeconomic sectors of society need to adapt to extreme weather and climate events (Adnan et al., 2017; Manzoor et al., 2022; Saleem et al., 2022). Changes in annual mean temperatures are found larger for most of the AEZs except Wet Mountains, Northern Dry Mountains, and Western Dry Mountains (Figure S3a in Supporting Information S1). Whereas, precipitation variability is greater for the Barani‐Rainfall and Wet Mountains zones (Figure S3b in Supporting Information S1). The larger risks to society and economic activities, in particular, to the lower‐income groups in these AEZs, and the impacts could be significant (Qazlbash et al., 2021). Increases in temperature, especially during heatwaves, disproportionately affect low‐income communities due to lack of infrastructure (Abid et al., 2016; Rana et al., 2022). While, the high‐income communities generally have better resources, knowledge, and infrastructure to adapt to changing climate conditions. Investigations on the impacts of extreme‐high temperatures on mortality in Pakistan have led to a 27% increase in the death rates (Khan Barakzai & Burney, 2021). For instant, changes in precipitation patterns can impact agriculture and the livelihoods of smallholder farmers in Pakistan (Manzoor et al., 2022). Climate change impacts exacerbate existing socioeconomic inequalities among marginalized communities (S. U. Khan et al., 2023). Addressing the impacts of climate change, therefore, requires a holistic approach that considers these socioeconomic disparities for environmental justice.

## 5 Conclusions

Spatiotemporal trends in mean to extreme temperature and precipitation events across various AEZs of Pakistan provide valuable insights into the changing climate dynamics in the region. The AEZs (i.e., northern, northeastern, and southern Pakistan) reveal significant warmer (wetter) trends to extreme temperature (precipitation) events during 1979–2020. The possible mechanisms responsible for warming in the region are land‐atmosphere radiational processes (i.e., low‐level clouds, surface thermal and solar radiations). Whereas, the low‐level tropospheric convergence favors moisture transport across Pakistan AEZs.

The population in the AEZs of Pakistan is increasingly exposed to both mean and extreme climate events for the period of last 42 years. Overall, a two‐fold increase in population exposure to extreme temperature (precipitation) events is evident during the recent climate period of 2000–2020 (in contrast to the reference period of 1979–1999), while some AEZs show a larger increase in exposure. The relative changes in population exposure increase the most, 60%−90% for the Northern Irrigated Plain, Barani/Rainfall, and Wet Mountains and least, 20%–30% for the Sandy Desert, Northern Dry Mountains, and Dry Western Plateau. The effect of climate as a driver accounts for nearly half of the total change in population exposure (>50%) to extreme climate events over major AEZs. In conclusion, addressing the population exposure to mean and extreme climate events in the AEZs of Pakistan requires a multi‐faceted approach that combines adaptation, mitigation, and resilience‐building strategies. By prioritizing these measures, Pakistan can mitigate the adverse effects of climate change, protect agricultural livelihoods, and enhance the well‐being of its population in the face of a changing climate.

## Conflict of Interest

The authors declare no conflicts of interest relevant to this study.

## Supporting information

Supporting Information S1

## Data Availability

The daily temperature and precipitation data sets for research purposes can be obtained by contacting the Pakistan Meteorological Department https://www.pmd.gov.pk/en/contact-us.php. The ERA5 reanalysis data is available online at Hersbach et al. (2023). The population data used for the creation of manuscript is given in Frieler et al. (2017). The R scripts used to calculate climate indices can be found at https://github.com/ECCC-CDAS/RClimDex/(X. Zhang et al., 2018). The figures were made with the NCAR Command Language version 6.6.2 (Boulder, 2019).
