# Supplementary material for: Population Exposure Changes to Mean and Extreme Climate Events Over Pakistan and Associated Mechanisms
Source: Geohealth. 2023 Oct 25;7(10):e2023GH000887. doi: 10.1029/2023GH000887 (PMC10599709; doi:10.1029/2023GH000887)
Supplement: Supplementary file 1 — Supporting Information S1 [file GH2-7-e2023GH000887-s001.docx]

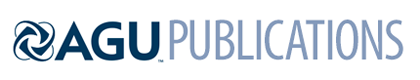


*GeoHealth*

Supporting Information for

**Population exposure changes to mean and extreme climate events over Pakistan and associated mechanisms**

**Farhan Saleem^1,2^, Wenxia Zhang^3^, Saadia Hina^4^, Xiaodong Zeng^1,2,5 *^, Irfan Ullah^6^, Tehmina Bibi^7^, Dike Victor Nnmadi^1^**

^1^ International Center for Climate and Environment Sciences, Institute of Atmospheric Physics, Chinese Academy of Sciences, Beijing 100029, PR China

^2^ College of Earth and Planetary Sciences, University of Chinese Academy of Sciences, Beijing 100049, PR China

^3^ State Key Laboratory of Numerical Modelling for Atmospheric Sciences and Geophysical Fluid Dynamics, Institute of Atmospheric Physics, Chinese Academy of Sciences, Beijing 100029, PR China

^4^ Department of Environmental Sciences, College of Agriculture and Environmental Sciences, Government College University Faisalabad 38000, Pakistan

^5^ Collaborative Innovation Center on Forecast and Evaluation of Meteorological Disasters, Nanjing University of Information Science and Technology, Nanjing 210044, PR China

^6^ College of Hydrology and Water Resources, Hohai University, Nanjing 210098, PR China

^7^ Institute of Geology, University of Azad Jammu and Kashmir, Muzaffarabad 33100, Pakistan

* corresponding author:

Xiaodong Zeng ([xdzeng@mail.iap.ac.cn](mailto:xdzeng@mail.iap.ac.cn))

**Contents of this file**

Text S1 to S4

Figures S1 to S3

Table S1

**S1.** Topographic and climatological features of AEZs in Pakistan

The zone of Indus Delta has arid tropical marine climate, and the daily mean Tmax and Tmin temperatures are 33 and 21 ºC with the daily rainfall amount of 1.5 mm. Clayey and silty soil conditions prevail in this zone and the main food crops here are rice, sugarcane, cotton and wheat. Climate is arid subtropical continental in the zone of Southern Irrigated Plain. While, the daily average Tmax and Tmin temperatures are around 35 and 19 ºC, and the daily rainfall total is 1.4 mm, respectively. The existing soils are calcareous silty and sandy loams, and the major crops of the zone include cotton, wheat, berseem and sorghum. Sandy Deserts zone has arid climate with dry and hot weather conditions. The reported daily extreme Tmax and Tmin temperatures are around 33 and 18 ºC, and the daily rainfall amount is 2.4 mm. Sandy and loamy soils exist in this zone; and the main land-use is grazing with shrubs, grasses and small plants. In the zone of Northern Irrigated Plain, the prevailing climatic patterns are semiarid. The daily recorded upper Tmax and lower Tmin temperatures are 32 and 17 ºC, and the daily rainfall amount is equals to 4.6 mm. The eastern and southern part consists of calcareous silt and loamy soils. The main food crops include wheat, rice, cotton and millet, while citrus and mangoes are the major fruit crops of this zone. Barani/ Rainfall zone occupies the salt range, Potowar Plateau, and foothills of Himalayan Piedmont. Weather is sub-humid; the daily Tmax and Tmin temperatures are about 30 and 16 ºC, with the daily rainfall total of 9.8 mm. Usual soils here are calcareous loams to silty loams. While, rice, maize, wheat, mustard and barley are the key crops here. The Wet Mountains zone covers the high mountains and narrow valleys. Climate is humid here; the daily temperature extremes of Tmax and Tmin are 26 and 13 ºC, and the daily rainfall sum is 16.7 mm. The soil of this zone composes of silty loam to silty clay; 25% of the area are under the rainfed crops (wheat and maize); while, the rest of the area are covered with forests. Weather in the zone of Northern Dry Mountains is subhumid. The daily high Tmax and low Tmin temperatures are around 24 and 10 ºC, with the daily rainfall equals to 4.0 mm. Clayey soil conditions exist on mountainous valleys and plains. The key land-use pattern here is grazing. The semiarid climatic conditions exist in the zone of Western Dry Mountains. The daily Tmax and Tmin temperature extremes are 25 and 10 ºC, and the daily rainfall total is about to 5.4 mm. The existing soils are loamy and calcareous in the valleys. The major landuse is grazing; wheat is grown with floodwater; while, apple, peach, plum, apricot and grapes are the major fruit crops of this zone. Weather and climate conditions are arid tropical marine in the zone of Dry Western Plateau. The upper and lower temperature thresholds are about 32 and 18 ºC, with the daily rainfall totals of 1.3 mm. Soils in plains are silty loams and calcareous. This zone mainly comprises xerophytic vegetation, grasses, small trees and wild olives, respectively. The last zone (Suleiman Piedmont) comprises piedmont plains of Suleiman Range. Climate is arid subtropical continental; the daily mean high and low temperatures are 33 and 18 ºC, and the daily rainfall sum is about to 2.4 mm, respectively. Soils are loamy and clayey in the zone, torrent watered is the primary land-use here, and wheat and millet are the common agriculture crops here (PARC 1980, Saleem et al. 2021).

**S2.** Serial correlation analysis

The presence of a serial correlation or auto correlation in the time series data may lead to the false detection of a trend. Prewhitening test is used to eliminate the effect of serial correlation in timeseries data (Figure S1). The degree of lag-1 autocorrelation was first tested on minimum, maximum temperatures and precipitation time series before applying a nonparametric Mann Kendall (MK) test (Kendall 1955, Mann 1945) and Sen's slope estimator method (Sen 1968). When the time series of indices did not depict a significant autocorrelation, the original MK test was applied to detect the trend. Several studies have used this method in literature (Yue &Wang 2002).

**S3.** Analysis of large-scale atmospheric patterns

The high pressure over Northeastern Pakistan can weaken the South Asian subtropical upper-level westerly jet (Figure S2a), causing a significant upper-level convergence over Pakistan (Figure S2b). Due to the convergence of upper-level airflow over Pakistan, the mass continuity produces a compensating downward flow. Consequently, a significantly sinking motion trend can be observed for the region (Figure S2c), which can further reduce low-level clouds (Figure 2g). Furthermore, the reduced cloud cover favors downwards shortwave radiation (Figure 2i), which upsurges the land surface temperature. Besides this, the significant warming trends of nighttime surface thermal radiation from the land surface may further warm the overlying atmosphere over Pakistan (Figure 2h).

**S4.** Temperature and precipitation variability across AEZs

Temperature variability and changes are found larger for most of the AEZs except Wet Mountains, Northern Dry Mountains, and Western Dry Mountains (Figure S3a). Whereas, precipitation variability and changes are greater for the Barani-Rainfall and Wet Mountains zones (Figure S3b). The climatic risks to population particularly, the low-income communities living in these AEZs become higher and the impact could be significant.


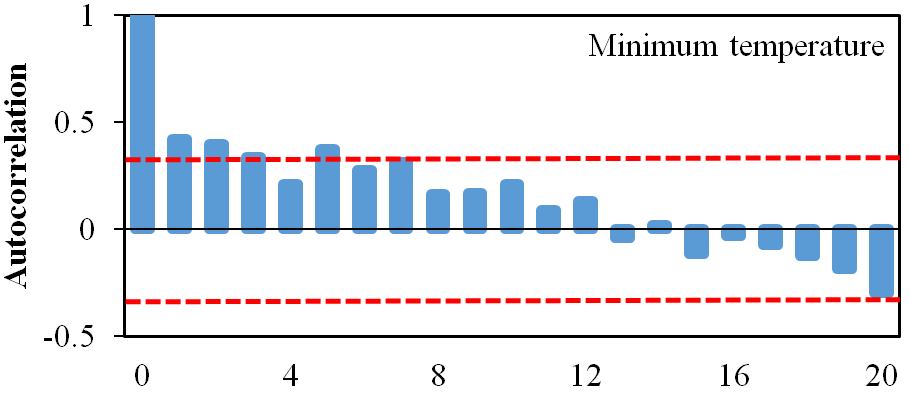

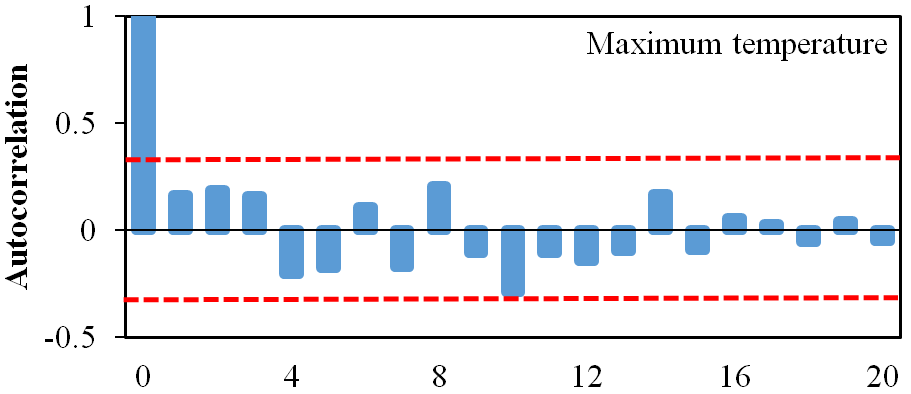

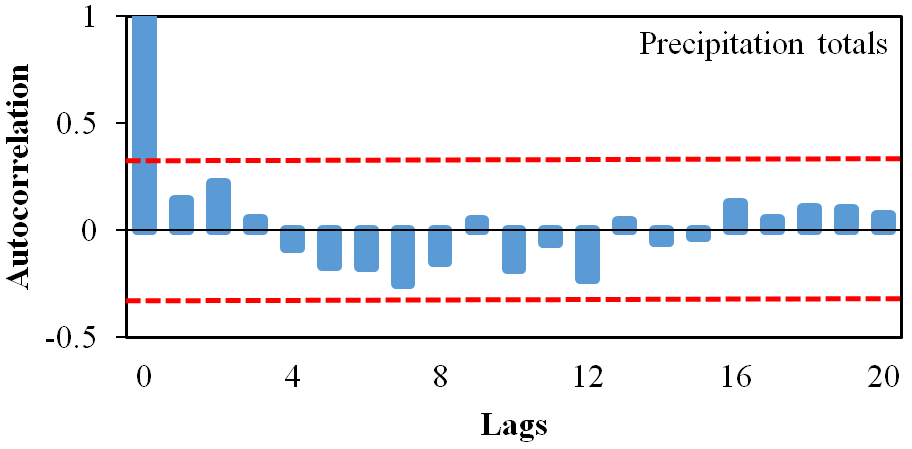


**Figure S1.** Autocorrelation analysis of minimum, maximum temperatures and precipitation totals on annual timescale under different lags. The dashed horizontal lines (red-color) on each panel plot show a confidence interval of 0.05 level.


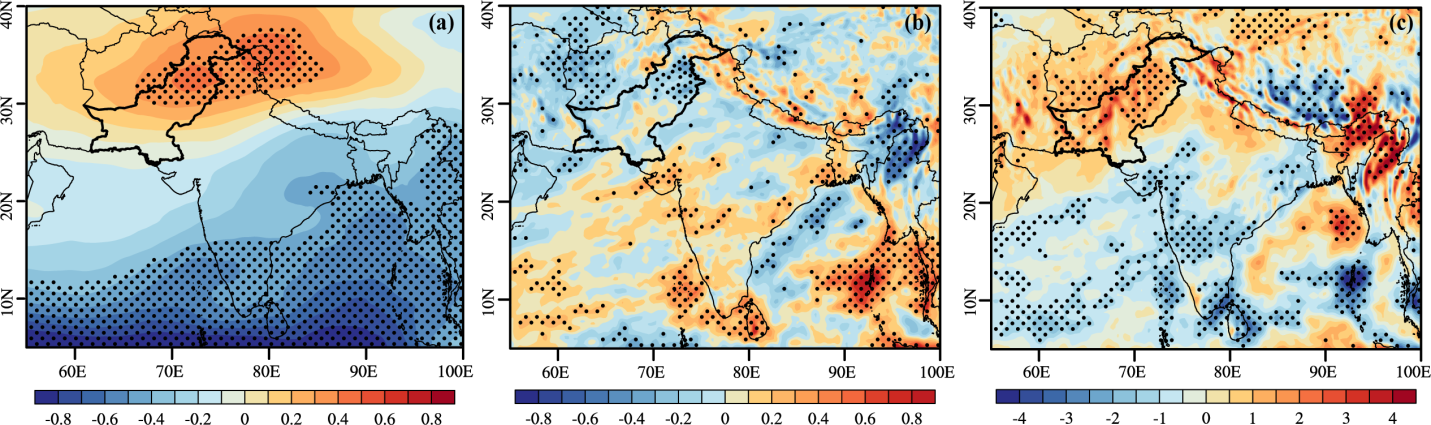


**Figure S2.** Trends of anomalous (a) 200h hPa zonal wind (m s^-1^), (b) 200 hPa relative divergence (10^-7^ s^-1^), and (c) 200 hPa vertical velocity (10^-3^ Pa s^-1^) respectively. The black dots highlight the areas significant at 95% level, calculated using Student’s *t*-test.


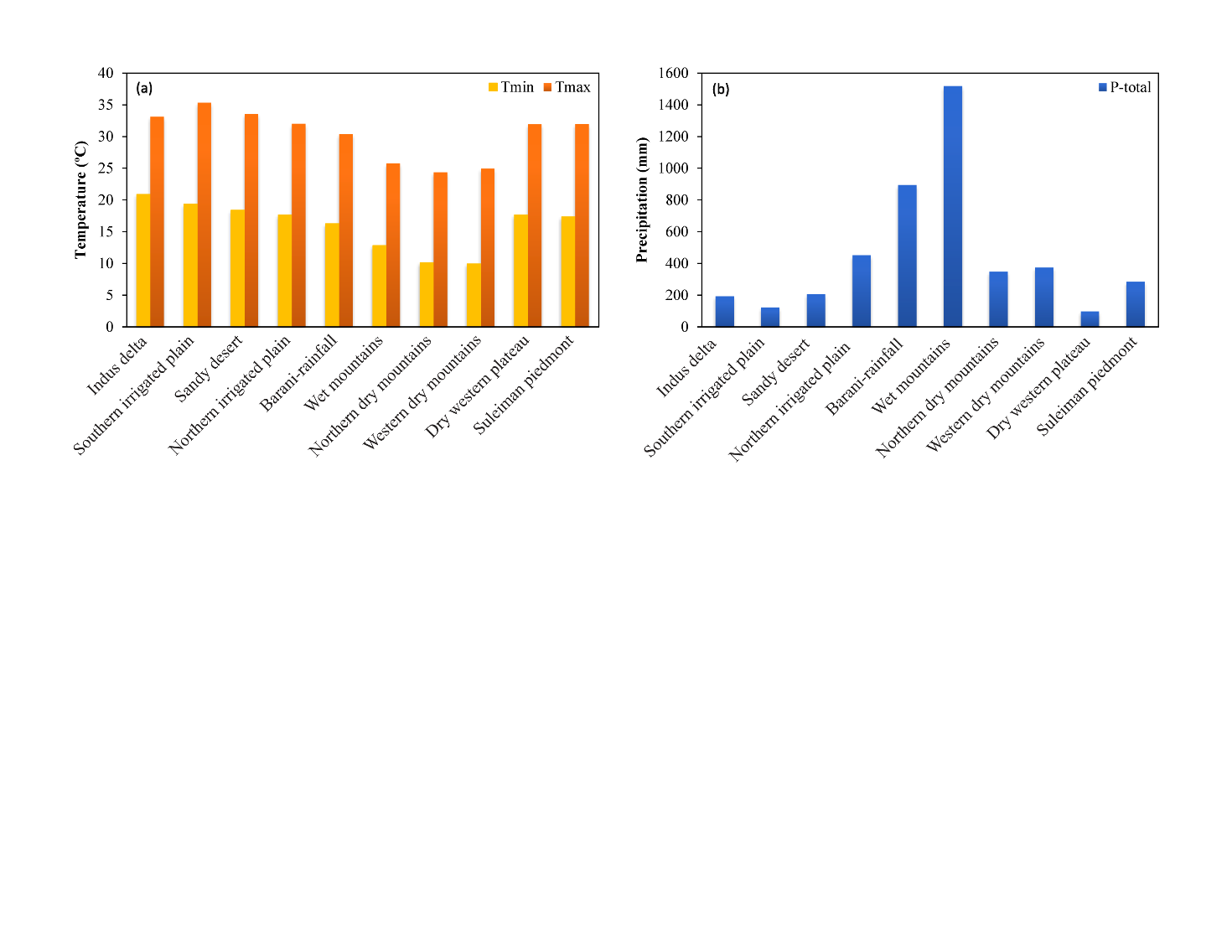


**Figure S3.** Changes in temperature and precipitation variability on annual timescale across Pakistan agro-ecological zones during the analysis period of 1979−2020.

**Table S1.** Pakistan agro-ecological zones their aridity index; the list of stations with geographical coordinates, and the regional mean temperature and rainfall patterns during 1979−2020.

| Agro-Ecological Zones  (Aridity Index) | Stations  (Names) | Stations  (Abb.) | Elevation  (m) | Latitude  (ºN) | Longitude  (ºE) | Temp  (ºC) | Prcp  (mm) |
| --- | --- | --- | --- | --- | --- | --- | --- |
| Indus Delta  (Arid, 0−10) | Badin | BDN | 0009 | 24.38 | 68.54 | 27.77 | 244.4 |
|  | Karachi | KHI | 0022 | 24.54 | 66.56 | 27.82 | 205.5 |
|  | Hyderabad | HYD | 0028 | 25.23 | 68.25 | 28.11 | 176.1 |
| Southern Irrigated Plain  (Arid, 0−10) | Nawabshah | NWB | 0037 | 26.15 | 68.22 | 28.28 | 128.4 |
|  | Padidan | PDD | 0046 | 26.51 | 68.08 | 28.31 | 122.2 |
|  | Jacobabad | JCB | 0055 | 28.18 | 68.28 | 28.59 | 149.2 |
|  | Rohri | ROH | 0066 | 27.40 | 68.54 | 28.05 | 114.8 |
| Sandy Desert  (Arid, 0−10) | Chhor | CHR | 0005 | 29.53 | 69.43 | 28.04 | 244.5 |
|  | Khanpur | KHN | 0088 | 28.39 | 70.41 | 27.10 | 125.3 |
|  | Bahawalpur | BPR | 0110 | 29.20 | 71.47 | 26.06 | 177.1 |
|  | Bahawalnagar | BGR | 0161 | 29.20 | 73.51 | 26.61 | 243.3 |
| Northern Irrigated Plain  (Semiarid, 15−24) | Multan | MUL | 0122 | 30.20 | 71.43 | 26.55 | 212.6 |
|  | Faisalabad | FSD | 0186 | 31.26 | 73.08 | 25.85 | 397.8 |
|  | Sargodha | SAR | 0187 | 32.16 | 72.89 | 25.22 | 481.9 |
|  | Lahore | LHE | 0214 | 31.35 | 74.24 | 26.59 | 689.7 |
| Barani **/** Rainfall  (Wet Sub-Humid, 30−35) | Mianwali | MWL | 0210 | 31.53 | 73.60 | 25.15 | 580.8 |
|  | Sialkot | SKT | 0255 | 32.31 | 74.32 | 23.98 | 980.5 |
|  | Jhelum | JLM | 0287 | 32.56 | 73.44 | 25.07 | 920.2 |
|  | Islamabad | ISL | 0540 | 33.68 | 73.04 | 21.70 | 1241 |
| Wet Mountains  (Humid, 35−55) | Kotli | KOT | 0614 | 33.31 | 73.54 | 22.19 | 1200 |
|  | Muzaffarabad | MUZ | 0702 | 34.22 | 73.29 | 20.11 | 1510 |
|  | Balakot | BKT | 0955 | 34.79 | 73.45 | 19.17 | 1598 |
|  | Murree | MUR | 2291 | 33.90 | 73.39 | 14.13 | 1770 |
| Northern Dry Mountains  (Dry Sub-Humid, 24−30) | Peshawar | PWR | 0327 | 34.11 | 71.61 | 22.40 | 488.2 |
|  | Gilgit | GIL | 1460 | 35.55 | 74.20 | 15.98 | 122.8 |
|  | Drosh | DRO | 1463 | 35.34 | 71.47 | 16.75 | 572.9 |
|  | Skardu | SKD | 2317 | 35.18 | 75.41 | 11.47 | 230.2 |
| Western Dry Mountains  (Semiarid, 15−24) | Khuzdar | KZD | 1231 | 27.50 | 66.38 | 23.38 | 266.9 |
|  | Zhob | ZOB | 1405 | 31.34 | 69.44 | 20.58 | 277.9 |
|  | Quetta | QTA | 1719 | 30.18 | 66.99 | 17.63 | 272.3 |
|  | Parachinar | PAR | 1725 | 33.52 | 70.05 | 16.68 | 895.1 |
|  | Kalat | KLT | 2015 | 29.03 | 66.58 | 14.96 | 214.8 |
| Dry Western Plateau  (Arid, 0−10) | Pasni | PAS | 0009 | 25.16 | 63.29 | 25.62 | 104.1 |
|  | Lasbella | LSB | 0087 | 26.14 | 66.10 | 28.40 | 182.5 |
|  | Nokkundi | NOK | 0682 | 28.82 | 62.75 | 26.11 | 33.54 |
|  | Dalbadin | DAL | 0848 | 28.53 | 64.24 | 23.92 | 92.98 |
|  | Panjgur | PNJ | 0968 | 26.58 | 64.06 | 24.83 | 107.2 |
| Suleiman Piedmont  (Arid, 0−10) | Sibi | SBI | 0133 | 29.53 | 67.87 | 25.53 | 177.5 |
|  | Dera Ismail Khan | DIK | 0171 | 31.55 | 70.52 | 27.59 | 316.5 |
|  | Barkhan | BKN | 1097 | 29.92 | 69.74 | 22.76 | 402.1 |

Aridity index values were calculated using De Martonne’s approach (de Martonne 1926).

**References**

de Martonne, E. (1926). Une nouvelle function climatologique: L'indice d'aridité. *Meteorologie, 2*, 449-459.

Kendall, M. G. (1955). Rank correlation methods. 1955. *Griffin*, *London*.

Mann, H. B. (1945). Nonparametric tests against trend. Econometrica. *Journal of the econometric society*, 245-259

PARC. (1980). Agro‐ecological Regions of Pakistan. *Pakistan Agriculture Research Council Islamabad.*

Saleem, F., Zeng, X., Hina, S., & Omer, A. (2021). Regional changes in extreme temperature records over Pakistan and their relation to Pacific variability. *Atmospheric Research, 250*, 105407.

Sen, P. K. (1968). Estimates of the regression coefficient based on Kendall's tau. *Journal of the American statistical association, 63*, 1379-1389.

Yue, S., & Wang, C. Y. (2002). Applicability of prewhitening to eliminate the influence of serial correlation on the Mann‐Kendall test. *Water resources research, 38*, 4-1-4-7.
